# Associations of activity, sedentary and sleep behaviors with oral health indictors in children and adolescents: a cross-sectional analysis

**DOI:** 10.1186/s44167-024-00057-5

**Published:** 2024-07-29

**Authors:** Ryan D. Burns, Hayley Almes, You Fu

**Affiliations:** 1 Department of Health and Kinesiology, University of Utah, 1850 East 250 South Room 237-D, Salt Lake City, UT 84112 USA; 2 School of Public Health, University of Nevada, Reno, NV USA

**Keywords:** Adolescent, Child, Exercise, Oral health, Screen time, Sleep

## Abstract

**Background:**

The purpose of this study was to examine the associations of physical activity (PA), recreational screen time, and sleep with indicators of poor oral health in youth.

**Methods:**

Participants were children and adolescents whose parents completed the 2022 National Survey of Children’s Health (*N* = 34,342; 49% female; Mean age = 11.9 +/- 3.5 years). The dependent variables were three binary response items that indicated the presence of toothaches, bleeding gums, and cavities within the previous 12 months. Independent variables were three items indicating the weekly frequency of 60 min of PA, hours of recreational screen time, and hours of weeknight sleep. Relationships between variables were examined using double-selection logistic regression with demographic, dietary, oral hygiene, and dental service covariates selected using the plug-in method of the least absolute shrinkage and selection operator.

**Results:**

Compared to zero days of PA, 4–6 days of PA per week was associated with 30% lower odds of bleeding gums (*p* = 0.021). Compared to less than 1 h of recreational screen time, 2 h or more hours of recreational screen time were associated with a 1.26 to 1.62 times higher odds of cavities (*p* < 0.01). Compared to 5 h or less of sleep, 7 to 10 h of sleep was associated with 47–61% lower odds of bleeding gums and 31–47% lower odds of cavities (*p* < 0.01). Secondary analyses indicated that at least 2 of the movement behavior recommendations had to have been adhered to for positive associations with indicators of oral health to be observed. For toothaches, bleeding gums, and cavities, meeting 2 or 3 recommendations was associated with lower odds of poor oral health; whereas adhering to only one recommendation was not.

**Conclusion:**

Children with higher PA and sleep durations had improved oral health indicators and children with longer periods of screen time exposure had poorer oral health indicators. Our findings recommend adhering to multiple movement behavior recommendations to achieve improvements in oral health indicators.

**Supplementary Information:**

The online version contains supplementary material available at 10.1186/s44167-024-00057-5.

## Introduction

Poor oral health is a public health issue that is often neglected in behavioral research [1]. Poor oral health in youth associates with jaw pain and discomfort, school absenteeism, school missed days, social stigma, and an overall low quality of life [2–4]. Preventive strategies such as tooth brushing, flossing, and regular dental cleanings and checkups greatly reduce the risk of oral health issues [5–7]. However, in adults, other behavioral strategies have been found to associate with lower risk of poor oral health [8, 9]. Regular amounts of physical activity (PA) and healthy durations of sleep have been linked to better oral health [10–12]. Mechanisms for these beneficial associations may include reduced local and systemic inflammation markers, better blood glucose control, and healthier diets [13–15]. Unfortunately, these associations have been unexamined in pediatric population.

PA, screen time, and sleep are time use behaviors that form a composition across a 24-hour day [16, 17]. Numerous studies have shown the health benefits of higher levels of PA, limited recreational screen time, and healthy sleep durations in youth [18–20]. Current recommendations include an average of 60 min of PA per day across the week including at least 3 days of muscle strengthening activity, limiting recreational screen time to 2 h or less, and receiving 8–11 h of sleep for children and 7–10 h for adolescents [21, 22]. The associations of adhering to these recommendations with oral health indicators are unknown. This information may help provide evidence of supplemental behavioral strategies that can be used to facilitate better oral health in youth. Given that poor oral health in childhood may track into adulthood and affect chronic disease risk, there is potential for greater impact. Therefore, the purpose of this study was to examine the associations of PA frequency, recreational screen time, and sleep duration with indicators of poor oral health in a large population-based sample of children and adolescents.

## Methods

### National survey of children’s health (NSCH)

The NSCH was designed to produce national and state-level data on the health of children aged 0–17 years old within the United States [23, 24]. The 2022 NSCH used an address-based sample selected from an extract of the Census Bureau’s Master Address File covering the 50 states and the District of Columbia [23, 24]. The addresses within each state were first sorted by strata, then organized into two groups by the block group poverty rate to ensure states had proportional representation of addresses in high poverty areas. Selected households received a mailed invitation that asked an adult (parent/guardian) who was familiar with children within the household to complete an online or paper screener questionnaire [23, 24]. If any child(ren) were identified from the screener questionnaire, the adult was directed to complete a topical questionnaire for one randomly selected child. Both the screener and topical questionnaires were available in both English and Spanish. For the 2022 NSCH, 54,103 surveys were completed for children between aged between 0 and 17 years old. The weighted overall response rate was 39.1%. Survey data were weighted to represent the population of non-institutionalized children ages 0–17 years old who live in housing units nationally and in each state [23, 24]. For the current study, the topical questionnaire for children and adolescents aged 6 to 17 years old was used for analysis.

### Data processing

The dependent variables were 3 items on the NSCH that asked about the child’s oral health; specifically, if they experienced toothaches, bleeding gums, or cavities in the past 12 months. The headers asked “During the past 12 months, has this child had frequent or chronic difficulty with any of the following? Toothaches. Bleeding Gums. Decayed teeth or cavities.”. Each item had a 1 = Yes and 2 = No response that was recoded to a binary variable for analysis using “No” as the referent (1 = Yes, 0 = No).

The primary independent variables were NSCH items asking about PA frequency, hours of recreational screen time per weekday, and hours of sleep per weeknight. The PA frequency item asked, “During the past week, on how many days did this child exercise, play a sport, or participate in physical activity for at least 60 minutes?”, with response ranging from 1= “0 Days” to 4=“Every day”. The recreational screen time item asked, “On most weekdays, about how much time did this child spend in front of a TV, computer, cellphone or other electronic device watching programs, playing games, accessing the internet or using social media?”, with responses ranging from 1=“Less than 1 h” to 5=“4 or more hours”. The hours of sleep item asked, “During the past week, how many hours of sleep did this child get on most weeknights?”, with responses ranging from 1=“Less than 6 h” to 7=“11 or more hours”. The independent variables were analyzed independently and as an aggregate variable by summing the number of behavioral recommendations adhered to (i.e., 0 to 3 recommendations).

Several control variables were entered into the analytic models to account for potential confounding influences. The least absolute shrinkage and selection operator (lasso; see statistical analysis section) was used to select from several potential control variables that were separate items of the 2022 NSCH. The potential control variables consisted of items related to participant age, sex, race, ethnicity, family income, body mass index classification, highest education of the parent, oral hygiene, dental visit and checkup frequency, preventative doctor visit frequency, health insurance coverage, and dietary items (i.e., sugary drinks, vegetable consumption).

### Statistical analysis

The YRBS complex survey design was accounted for using Stata’s “svy” prefix command. To examine the independent associations, multivariable double selection logistic regression models were employed. Logistic models were used to calculate adjusted odds ratios while adjusting for other movement behaviors and controls selected using the plug-in lasso method. Analyses were carried out using Stata’s “dslogit” command [25]. Lasso was incorporated into the analytic plan to account for potential omitted variable bias but avoid model overfitting [26, 27]. Separate models were run for each oral health dependent variables. The independent variables (PA, screen time, sleep) were used together within each of the models. An aggregate count variable representing the number of recommendations adhered to was also examined. Each model tested for effect modification by age, sex, race, ethnicity, family income, and body mass index classification using interaction terms. Because of the low prevalence of missing data (< 1.6% missing), complete case analyses were utilized. Sensitivity analyses using multiple imputation by chained equations across 10 imputation models verified use of complete case analysis as no differences were observed between methods. Alpha level was set at *p* < 0.05 and were carried out using Stata 18.0 statistical software package (Statacorp., College Station, Texas, USA).

## Results

### Descriptive statistics

There were a total of 34,362 total children and adolescents with parent-reported data (Mean age = 11.9 years +/- 3.5 years; 48.8% female). Missing data ranged from 0.4% for reporting of cavities to 1.6% for reporting of recreational screen time. Demographic data are reported in Table 1. Approximately, 3.3% of parents reported child toothaches, 1.5% reported bleeding gums, and 10.0% reported cavities within the previous 12 months. Parents reported that 11.8% of children had any oral health problems in the previous 12 months. Also, approximately 19.5% of parents reported that their child adhered to PA recommendations, 61.2% adhered to screen time recommendations, and 66.4% adhered to sleep duration recommendations. Approximately 9.5% of the sample adhered to all 3 movement recommendations, 33.3% to 2 recommendations, 40.0% to one recommendation, and 17.2% adhered to no recommendations. Missing data was less than 3% for all variables.

**Table 1:** Sample demographic and socioeconomic data

Variable	Group	Total Sample (*N* = 34,362)	Females (*n* = 16,532)	Males (*n* = 17,830)
Race	White	26,193 (69.9)	12,574 (69.5)	13,619 (70.2)
	Black/African American	2,570 (14.2)	1,262 (14.9)	1,300 (13.6)
	American Indian/Alaska Native	374 (1.4)	194 (1.5)	180 (1.2)
	Asian	2,223 (5.4)	1,089 (5.3)	1,134 (5.5)
	Native Hawaiian/Pacific Islander	227 (1.0)	100 (0.9)	127 (1.0)
	Two or More Races	2,775 (8.2)	1,313 (7.8)	1,462 (8.4)
Ethnicity	Not Hispanic/Latino	29,006 (73.2)	13,947 (73.3)	15,059 (73.0)
	Hispanic/Latino	5,356 (26.8)	2,585 (26.7)	2,771 (27.0)
Family Poverty Ratio	< 100%	4,603 (18.9)	2,244 (20.2)	2,359 (17.7)
	100–199%	5,669 (20.0)	2,664 (18.6)	3,005 (21.4)
	200–299%	5,431 (16.8)	2,643 (16.9)	2,788 (16.6)
	300–399%	4,535 (11.9)	2,150 (11.3)	2,385 (12.4)
	400% or more	14,124 (32.4)	6,831 (33.0)	7,293 (31.9)
BMI Class	< 5th %tile	2,831 (9.3)	1,245 (8.6)	1,586 (9.9)
	5th − 85th %tile	20,293 (58.1)	10,345 (61.6)	9,948 (54.6)
	85th − 95th %tile	4,783 (15.0)	2,226 (14.6)	2,557 (15.1)
	≥ 95th %tile	4,992 (17.7)	2,009 (14.9)	2,983 (20.4)

### Independent associations with oral health indicators

Adjusted odds ratios (AORs) are reported in Table 2 for each oral health indicator. After controlling for covariates, compared to zero days of PA, 4–6 days of PA per week was associated with 30% lower odds of bleeding gums (AOR = 0.70, *p* = 0.021). Compared to less than 1 h of screen time, 2 h or more hours of screen time were associated with a 1.26 to 1.62 times higher odds of cavities (AOR range = 1.26–1.62, *p* < 0.01). Compared to 5 h or less of sleep, 7 to 10 h of sleep was associated with 47–61% lower odds of bleeding gums (AOR range = 0.39–0.53, *p* < 0.01) and 31–47% lower odds of cavities (AOR range = 0.53–0.69, *p* < 0.01).

**Table 2:** Results from the double selection logistic regression analysis for each oral health indicator

		Toothache	Bleeding Gums	Cavities
		AOR (95% CI)	AOR (95% CI)	AOR (95% CI)
Independent Variable	Group			
Physical Activity (ref. = 0 days)	1–3 days	0.86 (0.69–1.07)	0.92 (0.71–1.18)	0.94 (0.82–1.07)
	4–6 days	0.78 (0.61–1.00)	**0.70** ^*^ **(0.52–0.94)**	0.95 (0.82–1.10)
	Every day	1.01 (0.79–1.31)	0.72 (0.51–1.01)	0.93 (0.80–1.10)
Screentime (ref. = Less than 1 h)	1 h	0.99 (0.70–1.40)	0.81 (0.49–1.32)	1.04 (0.85–1.26)
	2 h	1.09 (0.79–1.51)	0.79 (0.51–1.24)	**1.26** ^**^ **(1.05–1.51)**
	3 h	1.21 (0.86–1.71)	1.04 (0.67–1.64)	**1.42** ^***^ **(1.17–1.69)**
	4 h or more	1.29 (0.91–1.80)	1.32 (0.85–2.06)	**1.62** ^***^ **(1.34–1.95)**
Sleep Duration (ref.= 5 h or less)	6 h	1.68 (0.56–2.91)	0.76 (0.44–1.30)	0.88 (0.62–1.25)
	7 h	0.95 (0.56–1.60)	**0.53** ^*^ **(0.32–0.88)**	**0.70** ^**^ **(0.50–0.95)**
	8 h	0.83 (0.51–1.38)	**0.45** ^***^ **(0.28–0.73)**	**0.65** ^**^ **(0.47–0.87)**
	9 h	0.88 (0.52–1.46)	**0.53** ^**^ **(0.23–0.67)**	**0.65** ^**^ **(0.47–0.87)**
	10 h	0.73 (0.43–1.27)	**0.39** ^***^ **(0.22–0.67)**	**0.53** ^***^ **(0.38–0.72)**
	11 h or more	0.78 (0.42–1.44)	0.67 (0.35–1.27)	**0.53** ^**^ **(0.35–0.77)**

Note: AOR stands for adjusted odds ratio; 95% CI stands for 95% Confidence Interval; ref. stands for referent level; odds ratios adjusted for demographic, dietary, dental service, and parental health covariates selected using the plug-in method of the least absolute shrinkage and selection operator; bold indicates statistical significance, ^*^p < 0.05, ^**^p < 0.01, ^***^p < 0.001.

### Aggregate associations with oral health indicators

**Fig. 1:**
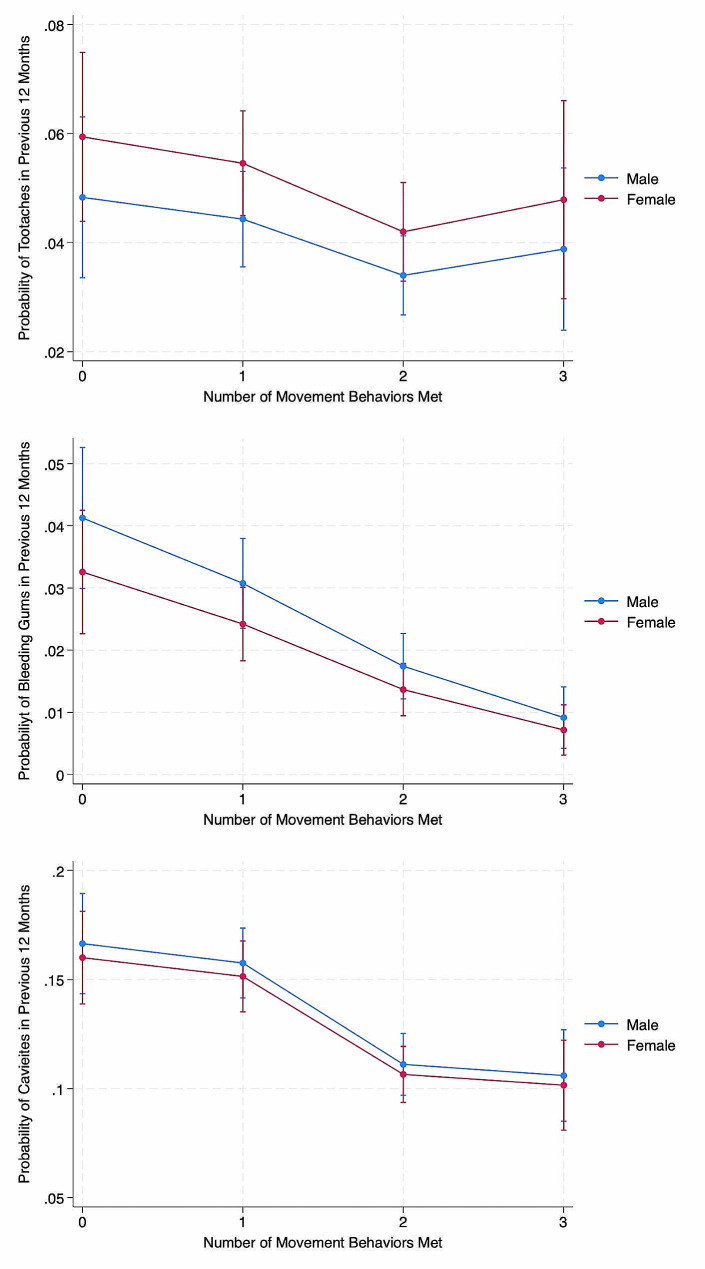
Predicted probabilities of having poor oral health as a function of adherence to the number of movement behavior recommendations

When analyzing data based on the number of movement recommendations adhered to, compared to adhering to 0 recommendations, adhering to 2 recommendations associated with 31% lower odds of toothaches (OR = 0.69, 95%CI:0.49–0.98, *p* = 0.036). Adhering to 2 recommendations (OR = 0.41, 95%CI: 0.27–0.61, *p* < 0.001) and 3 recommendations (OR = 0.21, 95%CI:0.12–0.39, *p* < 0.001) associated with a 59% and 79% lower odds of reported bleeding gums. Also, adhering to 2 recommendations (OR = 0.61, 95%CI: 0.50–0.75, *p* < 0.001) and 3 recommendations (OR = 0.58, 95%CI:0.44–0.77, *p* < 0.001) associated with 39% and 42% lower odds of reported cavities. Predicted probabilities, stratified by sex are depicted in Fig. 1. Finally, adhering to 2 movement behavior recommendations (OR = 0.59, 95%CI: 0.49–0.71, *p* < 0.001) and 3 recommendations (OR = 0.56, 95%CI:0.43–0.73, *p* < 0.001) associated with 41% and 44% lower odds of any poor oral health indicator. These associations were not significantly modified by child age, sex, race, ethnicity, family income, or body mass index. Predicted probabilities for these specific groups are presented in the Supplemental File.

## Discussion

The results indicated that: higher levels of PA were associated with lower odds of bleeding gums, higher durations of recreational screen time were associated with higher odds of cavities, and healthy sleep durations were associated with lower odds of bleeding gums and lower odds of cavities. Secondary analyses indicated that adhering to at least 2 movement behavior recommendations was associated with lower odds of toothaches, bleeding gums, and cavities.

The results from this study extend the findings observed in adults that higher levels of PA and healthy sleep durations are significantly associated with indicators of oral health in children and adolescents [28, 29]. Concerning PA, movement of higher intensities, frequencies, and durations may yield lower levels of both local and systemic inflammation in the body [30, 31], which results in lower risk of sickness and disease. Indeed, it is thought that the genesis of most chronic illness is local and/or systemic inflammation that can be partially mitigated by health behaviors [32]. The results of this study support this as 4 to 6 days of PA yielded significantly lower odds of bleeding gums. Bleeding gums is an indicator of local inflammation and is a primary indicator of periodontal gum disease [33, 34]. It is not clear why every day PA was not associated with oral health, but it may have to do with rest days during the week and possibly lower PA during religious or cultural days (e.g., on Sunday). Given that the current PA recommendation is an average of 60 min of PA per day throughout the week (rather than everyday PA), it can be stated that higher frequencies of PA may be beneficial for reducing odds of bleeding gums.

This is one of the first studies linking recreational screen time with oral health in a population-based sample of youth. The results indicated that higher amounts of recreational screen time was associated with higher odds of cavities. Dental caries or cavities partially results from the consumption of excess sugar and starch that lead to tooth decay and loss [35, 36]. Recreational screen time is often associated with consumption of snacks consisting of sugary drinks and foods [37, 38]. Excess screen time also takes time away from health enhancing PA; therefore, the combination of low levels of PA, reduced caloric expenditure and concurrent increase in caloric consumption in the form of excess refined foods and snacks during recreational screen time may be mechanisms that increase the odds of cavities for every hour of recreational screen time.

Sleep is a health behavior noted throughout the literature to have beneficial impacts on health and wellness. Healthy sleep durations have been shown to improve PA participation the following day, improve caloric expenditure, improve blood glucose control, and improve inflammation markers [39–41]. These mechanisms could be why achieving a healthy sleep duration associated with lower odds of both bleeding gums and cavities. There is some work also suggesting that receiving the proper amounts of sleep associated with lower consumption of refrained and processed foods and beverages that may also link to poor oral health [42, 43].

An interesting finding from the secondary analyses of this study was that at least 2 of the movement behavior recommendations had to have been adhered to for positive associations with indicators of oral health to be observed. For the outcomes of toothaches, bleeding gums, and cavities, meeting 2 or 3 recommendations was associated with lower odds of poor oral health; whereas adhering to only one recommendation was not. Therefore, multiple movement behaviors must be engaged in to observe a beneficial association with oral health indicators. This may be because indicators of oral health are determined by several mechanisms and that specific movement behaviors uniquely modulate these mechanisms. For instance, the reduction in the odds of cavities may be determined by both a reduction in systemic and local inflammation in addition to a reduction in the consumption of processed food. It could be that higher levels of PA or sleep lowers inflammation while reduction in recreational screen time may facilitate a lower consumption of processed and sugary foods. Thus, multiple behaviors are needed to target multiple potential mechanisms for poor oral health. Also, the observed association were not significantly moderated by age, sex, race/ethnicity, family income, or BMI classification. Therefore, adhering to the movement behavior recommendations may have a common beneficial impact across different pediatric populations.

Limitations of this research included the use of self-report methods that increase the risk of response bias. The overall response rate of the 2022 NSCH was relatively low at 39.1%, which may have increased the probability of selection bias. Use of device-based assessments for PA and sleep may have improved the internal validity of the findings. The study design was cross-sectional; therefore, the associations may also be bidirectional or have reverse causation. The NSCH is a parent report survey and thus the children were not directly asked about their behaviors or their oral health. Mechanisms of association were postulated but not directly examined. Therefore, it is not certain why movement behaviors associate with oral health indicators when accounting for good oral hygiene, dental check-ups, and professional cleanings.

In conclusion, PA, recreational screen time, and sleep duration were associated with indicators of oral health in children and adolescents. Adhering to at least 2 of the movement behavior recommendations is needed to detect a beneficial association. Additionally, these associations were not significantly moderated by age, sex, race/ethnicity, family income, or body mass index classification. Future research may want to explore the mechanisms of association such as local and systemic inflammation, blood glucose control, and clustering of other health behavior that facilitate healthy gums. This study provides evidence that engaging in higher levels of PA, limited recreational screen time, and healthy durations of sleep may supplement good oral hygiene to prevent oral health problems in children and adolescents.

## Electronic supplementary material

Below is the link to the electronic supplementary material.

Supplementary Material 1

## Data Availability

Data used in this study are publicly available at the US Census Bureau Website https://www.census.gov/programs-surveys/nsch/data/datasets.html.

## References

[1] Murtomaa H, Varenne B, Phantumvanit P, et al. Neglected epidemics: the role of oral public health to advance global health. J Glob Health. 2022;12:02001. 10.7189/jogh.12.02001. 35594383 10.7189/jogh.12.02001 PMC9122238

[2] Jackson SL, Vann WF Jr, Kotch JB, Pahel BT, Lee JY. Impact of poor oral health on children’s school attendance and performance. Am J Public Health. 2011;101(10):1900–6. 10.2105/AJPH.2010.200915. 21330579 10.2105/AJPH.2010.200915 PMC3222359

[3] Abreu MGL, Germano F, Antunes LS, Azeredo Alves Antunes L. Impact of oral health on the quality of life of preschoolers and their families. Glob Pediatr Health. 2021;8:2333794X21999145. 10.1177/2333794X21999145. 33796634 10.1177/2333794X21999145 PMC7983468

[4] Quadri MFA, Jaafari FRM, Mathmi NAA, et al. Impact of the poor oral health status of children on their families: an analytical cross-sectional study. Child (Basel). 2021;8(7):586. 10.3390/children8070586. 10.3390/children8070586 PMC8305805 34356565

[5] Thomson WM, Williams SM, Broadbent JM, Poulton R, Locker D. Long-term dental visiting patterns and adult oral health. J Dent Res. 2010;89(3):307–11. 10.1177/0022034509356779. 20093674 10.1177/0022034509356779 PMC2821461

[6] Marchesan JT, Byrd KM, Moss K, et al. Flossing is associated with improved oral health in older adults. J Dent Res. 2020;99(9):1047–53. 10.1177/0022034520916151. 32321349 10.1177/0022034520916151 PMC7375740

[7] Mattos-Silveira J, Matos-Lima BB, Oliveira TA, et al. Why do children and adolescents neglect dental flossing? Eur Arch Paediatr Dent. 2017;18(1):45–50. 10.1007/s40368-016-0266-4. 28138926 10.1007/s40368-016-0266-4

[8] Bawadi HA, Khader YS, Haroun TF, Al-Omari M, Tayyem RF. The association between periodontal disease, physical activity and healthy diet among adults in Jordan. J Periodontal Res. 2011;46(1):74–81. 10.1111/j.1600-0765.2010.01314.x. 20860591 10.1111/j.1600-0765.2010.01314.x

[9] Kurtović A, Talapko J, Bekić S, Škrlec I. The relationship between sleep, chronotype, and dental caries-a narrative review. Clocks Sleep. 2023;5(2):295–312. 10.3390/clockssleep5020023. 37218869 10.3390/clockssleep5020023 PMC10204555

[10] Movahed E, Moradi S, Mortezagholi B, et al. Investigating oral health among US adults with sleep disorder: a cross-sectional study. BMC Oral Health. 2023;23(1):996. 10.1186/s12903-023-03686-5. 38093226 10.1186/s12903-023-03686-5 PMC10720045

[11] Chan CCK, Chan AKY, Chu CH, Tsang YC. Physical activity as a modifiable risk factor for periodontal disease. Front Oral Health. 2023;4:1266462. 10.3389/froh.2023.1266462. 38024148 10.3389/froh.2023.1266462 PMC10679732

[12] Yoshimoto T, Hasegawa Y, Furihata M, et al. Effects of interval walking training on oral health status in middle-aged and older adults: a case-control study. Int J Environ Res Public Health. 2022;19(21):14465. 10.3390/ijerph192114465. 36361343 10.3390/ijerph192114465 PMC9657183

[13] Kimble R, McLellan G, Lennon LT, et al. Association between oral health markers and decline in muscle strength and physical performance in later life: longitudinal analyses of two prospective cohorts from the UK and the USA [published correction appears in Lancet Healthy Longev. Lancet Healthy Longev. 2022;3(11):e777–88. 10.1016/S2666-7568(22)00222-7. 36356627 10.1016/S2666-7568(22)00222-7 PMC10397540

[14] Hoppe CB, Oliveira JAP, Grecca FS, Haas AN, Gomes MS. Association between chronic oral inflammatory burden and physical fitness in males: a cross-sectional observational study. Int Endod J. 2017;50(8):740–9. 10.1111/iej.12686. 27578486 10.1111/iej.12686

[15] Holtfreter B, Stubbe B, Gläser S, et al. Periodontitis is related to exercise capacity: two cross-sectional studies. J Dent Res. 2021;100(8):824–32. 10.1177/0022034521995428. 33655783 10.1177/0022034521995428

[16] Tremblay MS, Carson V, Chaput JP, et al. Canadian 24-Hour movement guidelines for children and youth: an integration of physical activity, sedentary behaviour, and sleep. Appl Physiol Nutr Metab. 2016;41(6 Suppl 3):S311–27. 10.1139/apnm-2016-0151. 27306437 10.1139/apnm-2016-0151

[17] Dumuid D, Pedišić Ž, Stanford TE, et al. The compositional isotemporal substitution model: a method for estimating changes in a health outcome for reallocation of time between sleep, physical activity and sedentary behaviour. Stat Methods Med Res. 2019;28(3):846–57. 10.1177/0962280217737805. 29157152 10.1177/0962280217737805

[18] Fairclough SJ, Clifford L, Brown D, Tyler R. Characteristics of 24-hour movement behaviours and their associations with mental health in children and adolescents. J Act Sedentary Sleep Behav. 2023;2(1):11. 10.1186/s44167-023-00021-9. 38013786 10.1186/s44167-023-00021-9 PMC10234795

[19] Rollo S, Antsygina O, Tremblay MS. The whole day matters: understanding 24-hour movement guideline adherence and relationships with health indicators across the lifespan. J Sport Health Sci. 2020;9(6):493–510. 10.1016/j.jshs.2020.07.004. 32711156 10.1016/j.jshs.2020.07.004 PMC7749249

[20] Kim Y, Burns RD, Lee DC, Welk GJ. Associations of movement behaviors and body mass index: comparison between a report-based and monitor-based method using Compositional Data Analysis. Int J Obes (Lond). 2021;45(1):266–75. 10.1038/s41366-020-0638-z. 32661291 10.1038/s41366-020-0638-z PMC7752757

[21] Tremblay MS, Rollo S, Saunders TJ. Sedentary behavior research network members support new Canadian 24-Hour Movement Guideline recommendations. J Sport Health Sci. 2020;9(6):479–81. 10.1016/j.jshs.2020.09.012. 33071162 10.1016/j.jshs.2020.09.012 PMC7749241

[22] Wilhite K, Booker B, Huang BH, et al. Combinations of physical activity, sedentary behavior, and sleep duration and their associations with physical, psychological, and educational outcomes in children and adolescents: a systematic review. Am J Epidemiol. 2023;192(4):665–79. 10.1093/aje/kwac212. 36516992 10.1093/aje/kwac212 PMC10089066

[23] Child and Adolescent Health Measurement Initiative. Fast Facts: 2022 National Survey of Children’s Health. Data Resource Center for Child and Adolescent Health, supported by the U.S. Department of Health and Human Services, Health Resources and Services Administration (HRSA), Maternal and Child Health Bureau (MCHB). 2023a. Retrieved 12/01/23 from www.childhealthdata.org.

[24] Child and Adolescent Health Measurement Initiative. 2022 National Survey of Children’s Health: Methodology Report; 2023b. Retrieved 12/01/23 from www2.census.gov/programs-surveys/nsch/technical-documentation/methodology/2022-NSCH-Methodology-Report.pdf.

[25] Statacorp. Stata lasso reference manual: release 17. https://www.stata.com/manuals/lasso.pdf. [accessed 1 February 2023].

[26] Belloni A, Chernozhukov V, Wei Y. Post-selection inference for generalized linear models with many controls. J Bus Econ Stat. 2016;34:606–19. 10.1080/07350015.2016.1166116.

[27] Newcombe PJ, Connolly S, Seaman S, Richardson S, Sharp SJ. A two-step method for variable selection in the analysis of a case-cohort study. Int J Epidemiol. 2018;47(2):597–604. 10.1093/ije/dyx224. 29136145 10.1093/ije/dyx224 PMC5913627

[28] Sanchez GFL, Smith L, Koyanagi A, et al. Associations between self-reported physical activity and oral health: a cross-sectional analysis in 17,777 Spanish adults. Br Dent J. 2020;228(5):361–5. 10.1038/s41415-020-1306-3. 32170257 10.1038/s41415-020-1306-3

[29] Carra MC, Schmitt A, Thomas F, Danchin N, Pannier B, Bouchard P. Sleep disorders and oral health: a cross-sectional study. Clin Oral Investig. 2017;21(4):975–83. 10.1007/s00784-016-1851-y. 27178314 10.1007/s00784-016-1851-y

[30] Wang YH, Tan J, Zhou HH, Cao M, Zou Y. Long-term exercise training and inflammatory biomarkers in healthy subjects: a meta-analysis of randomized controlled trials. Front Psychol. 2023;14:1253329. 10.3389/fpsyg.2023.1253329. 37720640 10.3389/fpsyg.2023.1253329 PMC10499556

[31] Khalafi M, Symonds ME, Faramarzi M, Sharifmoradi K, Maleki AH, Rosenkranz SK. The effects of exercise training on inflammatory markers in children and adolescents: a systematic review and meta-analysis. Physiol Behav. 2024;278:114524. 10.1016/j.physbeh.2024.114524. 38521236 10.1016/j.physbeh.2024.114524

[32] Handschin C, Spiegelman BM. The role of exercise and PGC1alpha in inflammation and chronic disease. Nature. 2008;454(7203):463–9. 10.1038/nature07206. 18650917 10.1038/nature07206 PMC2587487

[33] Downing KF, Espinoza L, Oster ME, Farr SL. Preventive dental care and oral health of children and adolescents with and without heart conditions - United States, 2016–2019. MMWR Morb Mortal Wkly Rep. 2022;71(6):189–95. 10.15585/mmwr.mm7106a1. 35143467 10.15585/mmwr.mm7106a1 PMC8830625

[34] Goulão B, MacLennan GS, Ramsay CR. Have you had bleeding from your gums? Self-report to identify giNGival inflammation (the SING diagnostic accuracy and diagnostic model development study). J Clin Periodontol. 2021;48(7):919–28. 10.1111/jcpe.13455. 33751629 10.1111/jcpe.13455

[35] Chi DL, Scott JM. Added sugar and dental caries in children: a scientific update and future steps. Dent Clin North Am. 2019;63(1):17–33. 10.1016/j.cden.2018.08.003. 30447790 10.1016/j.cden.2018.08.003 PMC6242348

[36] Johansson I, Holgerson PL, Kressin NR, Nunn ME, Tanner AC. Snacking habits and caries in young children. Caries Res. 2010;44(5):421–30. 10.1159/000318569. 20720422 10.1159/000318569 PMC2969163

[37] Kelishadi R, Mozafarian N, Qorbani M, et al. Association between screen time and snack consumption in children and adolescents: the CASPIAN-IV study. J Pediatr Endocrinol Metab. 2017;30(2):211–9. 10.1515/jpem-2016-0312. 28099133 10.1515/jpem-2016-0312

[38] Nagata JM, Smith N, Alsamman S, et al. Association of physical activity and screen time with body mass index among US adolescents. JAMA Netw Open. 2023;6(2):e2255466. 10.1001/jamanetworkopen.2022.55466. 36757695 10.1001/jamanetworkopen.2022.55466 PMC9912127

[39] Sharma S, Kavuru M. Sleep and metabolism: an overview. Int J Endocrinol. 2010;2010:270832. 10.1155/2010/270832. 20811596 10.1155/2010/270832 PMC2929498

[40] Chaudhry BA, Brian MS, Morrell JS. The relationship between sleep duration and metabolic syndrome severity scores in emerging adults. Nutrients. 2023;15(4):1046. 10.3390/nu15041046. 36839404 10.3390/nu15041046 PMC9965711

[41] Irwin MR. Sleep and inflammation: partners in sickness and in health. Nat Rev Immunol. 2019;19(11):702–15. 10.1038/s41577-019-0190-z. 31289370 10.1038/s41577-019-0190-z

[42] Chapman CD, Nilsson EK, Nilsson VC, et al. Acute sleep deprivation increases food purchasing in men. Obes (Silver Spring). 2013;21(12):E555–60. 10.1002/oby.20579. 10.1002/oby.20579 23908148

[43] Knutson KL. Impact of sleep and sleep loss on glucose homeostasis and appetite regulation. Sleep Med Clin. 2007;2(2):187–97. 10.1016/j.jsmc.2007.03.004. 18516218 10.1016/j.jsmc.2007.03.004 PMC2084401

