# Supplementary material for: Associations of activity, sedentary and sleep behaviors with oral health indictors in children and adolescents: a cross-sectional analysis
Source: J Act Sedentary Sleep Behav. 2024 Jul 29;3:18. doi: 10.1186/s44167-024-00057-5 (PMC11960395; doi:10.1186/s44167-024-00057-5)
Supplement: Supplementary file 1 — Supplementary Material 1 [file 44167_2024_57_MOESM1_ESM.docx]

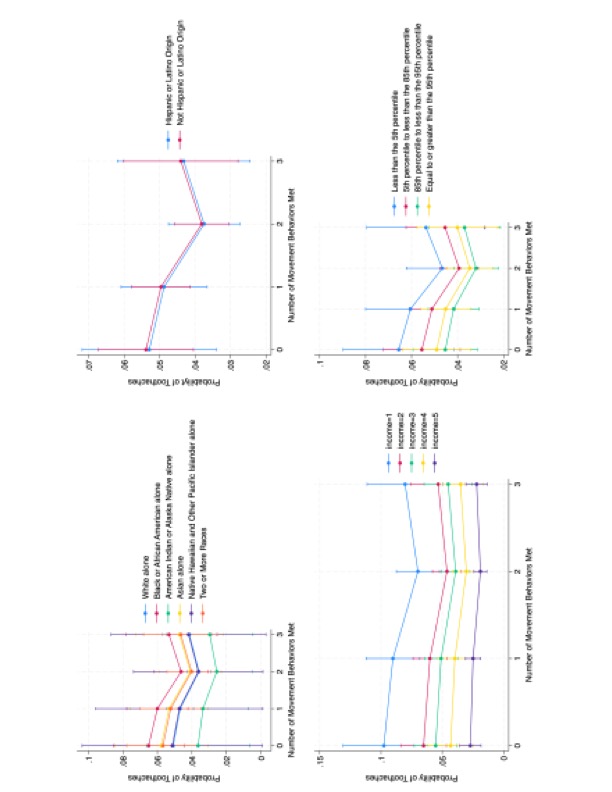


**Figure S1.** Predicted probability of toothaches in the past 12 months as a function of the number of movement behavior recommendation adhered to stratified by race (top left), ethnicity (top right), family income (bottom left), and body mass index (bottom right).


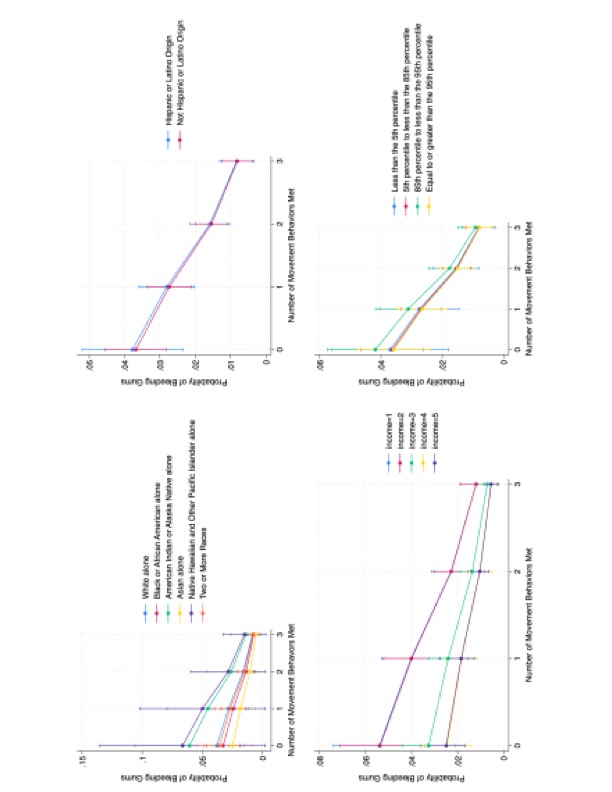


**Figure S2.** Predicted probability of bleeding gums in the past 12 months as a function of the number of movement behavior recommendation adhered to stratified by race (top left), ethnicity (top right), family income (bottom left), and body mass index (bottom right).


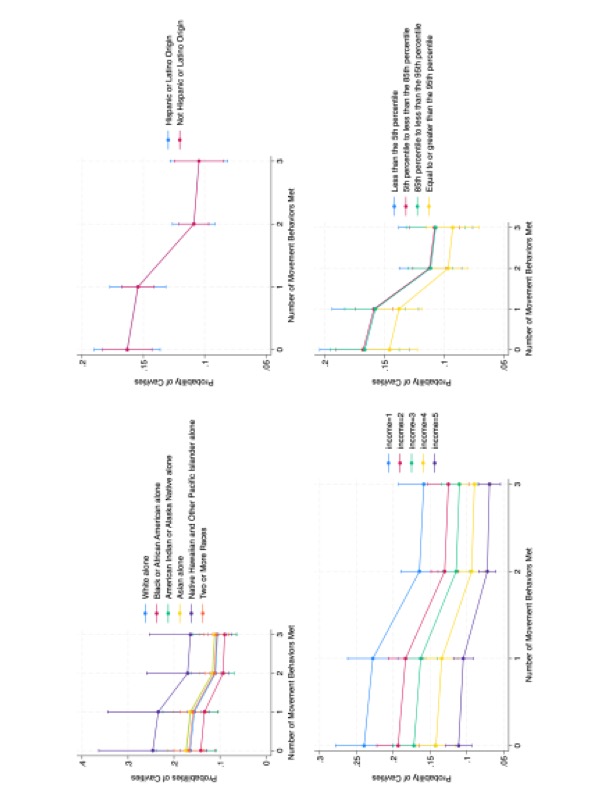


**Figure S3.** Predicted probability of cavities in the past 12 months as a function of the number of movement behavior recommendation adhered to stratified by race (top left), ethnicity (top right), family income (bottom left), and body mass index (bottom right).
